# Examining sustainability in a hospital setting: Case of smoking cessation

**DOI:** 10.1186/1748-5908-6-108

**Published:** 2011-09-14

**Authors:** Sharon Campbell, Karen Pieters, Kerri-Anne Mullen, Robin Reece, Robert D Reid

**Affiliations:** 1 Propel Centre for Population Health Impact, University of Waterloo, Waterloo, ON, Canada; 2 OMSC, Division of Prevention and Rehabilitation, University of Ottawa Heart Institute, Ottawa, ON, Canada; 3 Heart and Stroke Foundation of Ontario, Toronto, ON, Canada

## Abstract

**Background:**

The Ottawa Model of Smoking Cessation (OMSC) is a hospital-based smoking cessation program that is expanding across Canada. While the short-term effectiveness of hospital cessation programs has been documented, less is known about long-term sustainability. The purpose of this exploratory study was to understand how hospitals using the OMSC were addressing sustainability and determine if there were critical factors or issues that should be addressed as the program expanded.

**Methods:**

Six hospitals that differed on OMSC program activities (identify and document smokers, advise quitting, provide medication, and offer follow-up) were intentionally selected, and two key informants per hospital were interviewed using a semi-structured interview guide. Key informants were asked to reflect on the initial decision to implement the OMSC, the current implementation process, and perceived sustainability of the program. Qualitative analysis of the interview transcripts was conducted and themes related to problem definition, stakeholder influence, and program features emerged.

**Results:**

Sustainability was operationalized as higher performance of OMSC activities than at baseline. Factors identified in the literature as important for sustainability, such as program design, differences in implementation, organizational characteristics, and the community environment did not explain differences in program sustainability. Instead, key informants identified factors that reflected the interaction between how the health problem was defined by stakeholders, how priorities and concerns were addressed, features of the program itself, and fit within the hospital context and resources as being influential to the sustainability of the program.

**Conclusions:**

Applying a sustainability model to a hospital smoking cessation program allowed for an examination of how decisions made during implementation may impact sustainability. Examining these factors during implementation may provide insight into issues affecting program sustainability, and foster development of a sustainability plan. Based on this study, we suggest that sustainability plans should focus on enhancing interactions between the health problem, program features, and stakeholder influence.

## Background

Hospital care for smoking-related illnesses represents an important part of the healthcare burden. Smokers average more than twice as many hospital days compared to individuals who have never smoked [1]. There is overwhelming evidence that quitting smoking has beneficial effects on overall health and both acute and chronic disease outcomes [2-4]. Smoking cessation interventions provided to hospitalized smokers have been shown to improve smoking abstinence rates, along with healthcare utilization and surgical outcomes [5,6].

Numerous studies have examined the effectiveness of hospital smoking cessation programs [7]. However, few studies have examined the sustainability of these programs. In reviewing controlled studies of hospital inpatient smoking cessation programs, France *et al*. [8] contacted nine study authors to determine if the program was still operating. The authors found that no site had maintained a smoking cessation intervention to reach all hospitalized smokers; one site maintained a disease management program for secondary prevention of cardiac disease that includes counselling, and a second site provided smoking cessation intervention at the hospital through a consultation service, if the attending physician made a referral [8]. In another study, Taylor *et al*. [9] recruited six hospitals to participate in a study of the implementation and institutionalization (defined as less intensive involvement of the research team compared to the implementation phase) of an inpatient tobacco use cessation program. Of the five hospitals that reached the institutionalization phase, one hospital improved recruitment rates by hiring a full-time tobacco cessation expert, and a second met the target of 25% smoking abstinence at six months by increasing the number of follow-up calls per patient [9]. Smoker recruitment and quit rates decreased in all of the other hospitals [9]. The authors noted that constraints on financial and staff resources, lack of system supports for the recommended cessation activities, and the need for continued staff support and performance feedback were major barriers to institutionalization.

The dearth of studies about the sustainability of hospital-initiated cessation programs is unfortunate; these programs are feasible and effective at improving patient outcomes, but continuation beyond the implementation phase has not been consistently demonstrated. It is important to gain a better understanding of how programs become embedded into hospital operations to avoid losing the overall benefit that these programs have on the tobacco burden, hospitalizations, and health status of smokers.

The Ottawa Model for Smoking Cessation (OMSC), an inpatient smoking cessation program, was first developed for cardiac patients at an Ontario hospital in 2002 [10]. It consists of five activities: identify smokers on admission, document smoking status on patient record, provide identified smokers with advice and behavioural support with quitting, offer patients smoking cessation medications during their hospital stay, and offer follow-up support upon discharge to smokers who wish to quit. Follow-up is monitored by an automated, interactive voice response (IVR) system that tracks patients for up to six months [11]. Any patients experiencing difficulty quitting are then contacted by either University of Ottawa Heart Institute (UOHI) staff or hospital staff for continued support. The data collected by the IVR system also support performance monitoring and feedback for quality assurance purposes and demonstrate program impacts.

In 2006, additional funding allowed UOHI to implement the OMSC in other hospitals in Ontario. An absolute increase in long-term cessation rates of 11.1% (from 18.3% to 29.4%) was seen in the general hospital setting [12]. Given the effectiveness of the OMSC, further funding was provided in 2008 to expand the OMSC to seven other Canadian provinces [13]. The purpose of this evaluation was to understand how hospitals using the OMSC were addressing sustainability, and determine if there were critical factors that should be addressed before expansion across Canada.

### Conceptualizing sustainability

Sustainability is described by various authors as 'institutionalization,' 'incorporation,' 'maintenance,' and 'continuation' of a specific intervention over time, often after external funding has been reduced or withdrawn [14-21] (Table 1). O'Loughlin *et al*. investigated a national heart health promotion program to determine the permanence of different interventions [17]. Hanson *et al*. [22], examined differences in how stakeholders from three community demonstration projects conceptualized the sustainability of a fall prevention program. While the concepts of 'continuation, to maintain, to carry on' were common across community definitions, there were differences in defining what was to be sustained (the program itself or the expected health benefit) and how this would occur (*e.g*., with or without adaptation, through partnerships, institutionalization, or new funding) [22]. The authors concluded that different understandings of sustainability can affect perceptions of the overall success of the project [22].

**Table 1:** Definitions and conceptualizations of sustainability

First Author	Year	Definition/Conceptualization	Paper Details
Bracht [14]	1994	-sustainability is conceptualized as incorporation--'the maintenance of specific intervention program types over time, after external funding resources' (p.246)	-measured long-term program maintenance through annual surveys to assess the level of incorporation (*e.g*., who is operating program, program modifications) of 27 Heart Health intervention programs

Shediac-Rizkallah [16]	1998	-sustainability is likely a matter of degree rather than an 'all or none' phenomenon - definition must specify *what *is to be sustained, how or by whom, how much and by when	-presented an organizing framework for conceptualizing and measuring sustainability

O'Loughlin [17]	1998	-permanence: 'At this point in time, how permanent do you think the (intervention) is at (provider)?' (p.704)	-investigated factors related to the perceived sustainability of heart health promotion interventions

Greenhalgh [18]	2004	-'making an innovation routine until it reaches obsolescence.' (p.582)	-summarized an extensive literature review about sustaining innovations in health service delivery

Pluye [19]	2004	-sustainability is a parallel process that occurs at the same time as implementation - events can be specific to sustainability, specific to implementation, or belong to both sustainability and implementation	-reviewed empirical studies on program sustainability

Scheirer [15]	2005	-three definitions for sustainability: continued program activities; continued program benefits; maintained community capacity	-review of 19 empirical studies on sustainability of health-related programs in Canada and US

LaPelle [20]	2006	-defined levels of program sustainability (*i.e*., none, low, moderate, and high) based on the extent to which community-based tobacco treatment services were able to continue after program funding was terminated.	-used qualitative analysis of state and community level programs to investigate factors contributing to sustainability of services after defunding

Gruen [21]	2008	- the simplest definition of sustainability is the 'capability of being maintained at a certain rate or level' (p.1580).	-systematically reviewed conceptual frameworks and empirical studies about health program sustainability

Scheirer [15] suggests that ideally, sustainability would be defined in terms of continuing program activities that are necessary to obtain the intended outcome. Shediac-Rizkallah and Bone [16] go further and advise specifying 'what is to be sustained, how or by whom, how much and by when'. Gruen *et al*. [21] define sustainability as 'the capability of being maintained at a certain rate or level.' These authors recommend using a precise, measurable description of what constitutes sustainability. This approach makes it possible to separate interventions into sustainable and not sustainable, and investigate processes, barriers, and facilitators more accurately. Using a measurable definition of sustainability may also be helpful in discerning implementation and sustainability processes. Pluye *et al*. [19] suggest these occur together, making it difficult to define sustainability as a unique process.

**Figure 1:**
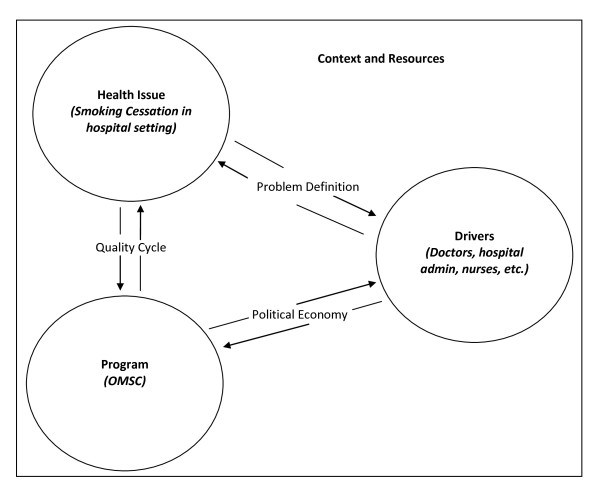
**Application of the OMSC to a Sustainability Model (Gruen RL, *et al*. Lancet 2008, 372:1579-1589.)**.

Knowledge of what affects sustainability can inform strategies to enhance the likelihood that interventions will continue after implementation. Shediac-Rizkallah and Bone [16] point to design characteristics of the program and the implementation process as important factors affecting sustainability. Shediac-Rizkallah and Bone [16] as well as Gruen *et al*. [21] also identify the organizational setting, culture, and community (*i.e*., political) environment as important factors that affect sustainability. Gruen *et al*. [21] propose an interactional model that describes links between the health problem, program intervention, and stakeholders (Figure 1). These links are presented as:

1. 'problem definition' - reflects interactions between the health issue and the drivers, and their perceptions that the health issue is important to their organization and fits with other priorities;

2. 'political economy' - describes interactions between the program and organizational drivers, and the degree of engagement or commitment drivers have for the program; and

3. 'quality cycle' - refers to the interactions between the health problem and the program, and the extent to which the program is able to demonstrate the expected impact on the health problem [21].

Hospital-based smoking cessation interventions like the OMSC can be effective in helping smokers quit, but long-term sustainability is required to improve health and healthcare utilization at the population level. As the OMSC is implemented in hospitals across the country, sustainability becomes critical. The purpose of this study was to understand how hospitals, which had already implemented the OMSC, were addressing sustainability. The findings of this study will be taken into consideration by the UOHI in their expansion plans.

## Methods

An evaluation advisory group, consisting of members from the UOHI, the Heart and Stroke Foundation Ontario, and a former OMSC nurse coordinator, provided input into the study design, conceptualization and definition of sustainability, development of the interview questions, and review of findings.

### Operational definition of sustainability

Sustainability of the OMSC was operationalized as the performance of all OMSC activities at the same or higher level than at the time of initial implementation (launch date). To achieve this, hospitals were asked to make OMSC activities part of normal hospital routine, accept responsibility to track performance, and provide performance feedback to the hospital cessation program, administrators, and staff.

### Hospital selection

UOHI identified 14 hospitals for possible inclusion in the study, and provided the evaluation team with the names and contact information of the smoking cessation coordinator (SCC) at each hospital. The evaluation team selected eight hospitals based on performance of OMSC activities (either higher or lower than baseline) and the date when the hospital began implementing the program. This study was reviewed by and received clearance from the Office of Research Ethics, University of Waterloo.

### Data collection

Hospitals (n = 8) were sent an introductory letter explaining the purpose of the study, followed by a telephone call one week later. Following hospital approval, information packages were mailed to the identified SCC who was asked to identify the hospital decision maker (DM) most familiar with the OMSC, explain the study, and get permission for the evaluation coordinator to contact the DM. The evaluation coordinator then scheduled and conducted individual semi-structured telephone interviews with the SCC and DM.

### Interview questions

The evaluation team developed a semi-structured interview (Additional File 1) that focused on: program implementation factors (*e.g*., why the hospital decided to implement the OMSC, how the OMSC was operating within the hospital, how challenges in implementation were handled); organizational setting (*e.g*., which units implemented the OMSC program); hospital reactions to the OMSC program, and perceived sustainability of the program (*e.g*., expected changes to the program, confidence that the program would be sustained, and challenges and barriers to sustaining the OMSC). The interview questions were reviewed by the advisory group and pilot tested with the former OMSC coordinator.

### Analysis

The interviews were audio-recorded, transcribed, and open coding was used to identify themes. Data from the DMs and SCCs were combined to reflect the perspective of the hospital. Two researchers discussed the coding and themes, resolving differences by consensus. Initially, results were organized by interview question, and a cross-comparative table was created to examine OMSC sustainability and program launch date, program location, and coordinator responsibilities (Additional File 2). However, as analysis proceeded, it became clear that the emerging themes fit with the types of interactions proposed by Gruen *et al*. [21]. This model was then used to organize and report the findings.

## Results

### Hospital recruitment and participation

Six of the eight selected hospitals (75%; 43% of the 14 eligible hospitals) agreed to participate. Of the two hospitals that declined, one was too busy and the other was unable to obtain hospital ethics approval in sufficient time to be included in the study. Time and budget limited the number of hospitals selected and the ability to replace hospitals that refused participation.

One DM and one SCC were interviewed at each participating hospital with two exceptions; we were unable to interview the DM at one hospital, and at another we interviewed two DMs at their request. Interviews lasted between 16 and 59 minutes (DM mean interview was 39 minutes; SCC mean interview was 47 minutes). Interviews were conducted between October 2009 and December 2009.

DMs held senior hospital administrative roles (*e.g*., director, clinical manager, chief nursing officer), and all were influential in bringing the OMSC to their hospital. The SCCs were unit nurses (n = 4), program manager (n = 1) and dedicated SCC (n = 1). One SCC had been involved in the initial implementation. Four SCCs had some dedicated time to educate staff and communicate program results, one was responsible for the IVR component only, and one did not have any unique responsibilities pertaining to the OMSC.

### Sustainability

#### Implementation and program design factors

Hospitals differed in how they implemented the OMSC (Additional File 2). Interestingly, we did not see clear differences in these factors between hospitals with sustainable and unsustainable OMSC programs as discussed below.

Three hospitals implemented the OMSC in general inpatient care units, and three selected special care units. Participating hospital units were selected based on staff interest, ability to redeploy resources and patient smoking rates. OMSC counselling was provided by nurses during routine care, by dedicated smoking cessation counsellors, or by specially trained nurses.

UOHI nurse specialists provide the IVR follow-up support to three hospitals. The other three hospitals are responsible for managing their own IVR, and have received funding for up to 1,000 patients. These hospitals provided differing perspectives on continuing patient follow-up with this system. Hospital E plans to continue IVR and is seeking funding. Hospital F does not plan to continue IVR follow-up due to funding concerns and frustrations with the software, and did not discuss alternative approaches to patient follow-up. Hospital C is unsure about the future of the IVR follow-up due to costs and questions the hospital's role in providing the IVR service, as opposed to connecting patients with a service in the community or a smoker's quit line.

All hospitals with a higher level of OMSC activity allocated a percentage of the SCC's time (range from 10% to 100%) to support the program (*e.g*., educate staff, ensure that patients are counselled, communicate program results). The two hospitals with lower than baseline OMSC activity either had not appointed a SCC or assigned the SCC to manage IVR follow-up only.

### Interactional themes

Themes that emerged from the interviews and qualitative analysis are presented below, along with the application to the OMSC. We found that applying the Gruen *et al*. model [21] and examining the interactions between the health problem (defined by UOHI as 'smoking by patients admitted to hospital'), the program (*i.e*., the OMSC activities), and program drivers (*e.g*., key stakeholders such as funders, managers, hospital administrators, policy makers, and community leaders), provided greater insight into the sustainability of the OMSC. These interactions and the likelihood of sustainability were influenced by the social, cultural, political, and economic context within each hospital setting (Figure 1). Application of Figure 1 to the OMSC is outlined below.

### Problem definition - how health concerns are identified and defined to meet the needs of people with influence

Key informants (*i.e*., SCCs and DMs) viewed smoking cessation as an important health issue that fit with the hospitals' corporate objectives of restoring health, or with the hospital's smoke-free property initiative:

'This is the number one type of prevention we can actually do for the top admitting diagnosis, so this is certainly going to affect our length of stays, better outcomes for patients.' (DM 3)

'I think it all comes down to patient health. How can a hospital not be tackling the number one killer?' (SCC 5)

'For years and years, healthcare workers, we made it okay for the public to smoke because we aren't saying that it's not okay. We weren't providing opportunities for them to see alternatives or how to help them, because it is an addiction, it is a disease. I think healthcare needs to lead the way.' (SCC 6)

Study respondents also expressed that addressing smoking cessation within the hospital faced some resistance. One DM relayed the attitude of the medical leadership, 'Is this something that really should fit into the hands of an acute care facility?' Another DM described how the hospital nurses felt the OMSC was an extra-burden on already busy staff. However, the DM believed patient tobacco use is an 'occupier of time':

'And it makes it easier for staff to just have their patients go out and smoke because for that period of time they don't have to deal with them. Rather than taking that time to say to the patient that we need to address your tobacco use as it matches your ability to recover from your medical condition, from your surgical condition, from your other conditions. We still don't have that. I don't think that we have it across a lot of healthcare. I don't think we are unique in that at [DM hospital].' (DM 5)

Staff behaviour began to change when nicotine replacement therapy (NRT) was made available as unit stock and the DM framed delaying the application of NRT as a medication error and patients smoking as a 'failure to treat their nicotine withdrawal.' (DM 5)

Several key informants suggested that framing hospital smoking cessation programs in terms of costs and benefits would influence decisions of the provincial Ministry of Health and Long Term Care to fund hospital cessation programs:

'If we take a very aggressive approach to addressing the use of tobacco in patients then we will have cost savings in our hospitals...we will have reduced days of stay, less infections. So I think that the approach, honestly, needs to talk about, obviously it's a wellness thing, and it's important, but hospital administrators are interested in the bottom line. They need to see this as an investment, not an expense. Because if you save two days of stay on the average length of stay, or even one day of stay for every patient who comes in who is a smoker, compared to patients who don't, who have the same procedure, I think that is very powerful data.' (DM 5)

### Political economy - how the program engages stakeholders

Although the four hospitals that implemented the OMSC using unit nurses did so for budgetary reasons, respondents at those hospitals felt that this approach helped embed the program into patient care and fostered sustainability by engaging frontline healthcare workers in the program:

'Nurses are used to healthcare teaching, so they see assessing patients' readiness [to quit smoking] as a good fit. It's amazing once they get committed at that point, how I think that's the sustainability component, because they are living and breathing it every day.' (DM 2)

A SCC with some dedicated time to counsel patients noted that other nurses had difficulty finding time to counsel patients:

'If [the other nurses] know I am coming in, perhaps they won't [deliver the program]; they will leave it for me. Because they don't have the hours dedicated to it, they have to try and fit it into their day and an assessment, the first counselling sessions take about a good 40 minutes or so by the time you are done the paperwork and that is a lot into their already busy day.' (SCC 2)

At another hospital, in order to engage nurses in the counselling process, the sessions were modified to take place when nurses are providing other care.

Two SCCs mentioned that their expectations about the program changed after seeing the effect it had on patients:

'But then when you find that the patient is less irritated, the patient is less restless, if you can provide them with some nicotine replacement and then you get one less problem to deal with, there's a benefit to it.' (SCC 6)

'I would say now, my emphasis is more to make them comfortable while they are in the hospital and hopefully they will [quit]. It is still in the long run to make them quit. However, when they are comfortable in the hospital and they see that they can go craving-free for a few days then that sort of gives them the courage to think about quitting or it teaches them that quitting can be an option.' (SCC 2)

### Strategies to engage stakeholders

#### Engaging champions

Two of the four hospitals with higher levels of OMSC activity mentioned that they used champions (*i.e*., individuals who promote the OMSC to hospital staff) to overcome staff resistance and gain acceptance of the model, thus promoting stakeholder engagement in the program. Hospitals strategically chose individuals with high credibility, enthusiasm about the program, and their passion for smoking cessation. One hospital, experiencing resistance from the medical leadership, enlisted a physician champion who 'made presentations and started to order medications for patients, to convince colleagues that it's a safe thing to do' (DM 1).

Some respondents also felt that program champions were necessary to keep the issue of smoking cessation on the hospital's 'front burner' amid competing priorities, to be able to add to the program, and to ensure that people comply with the program.

#### Supporting drivers

The program also engages drivers by providing them with support during the implementation phase. Respondents felt that the UOHI facilitator played a major role, 'She knew how everything should run and it was very, very new to us. She had all of the answers' (SCC 2). A hospital with a lower level of OMSC activity found that UOHI's feedback was helpful in providing input into problems they were experiencing:

'They would meet with us and look at how our audits were reporting, and looking at what some of our problems were, we were identifying how to improve and it was something that I thought was quite acceptable for new programs. You would troubleshoot as you went along.' (SCC 6)

Despite help with specific problems, the two hospitals with lower OMSC activity levels indicated that they did not always feel supported:

'Sometimes I don't feel supported. Sometimes I feel badgered. ...I think at this point we're feeling a little overwhelmed by what's before us.' (DM 5)

'Whenever there was a decrease in numbers, I'm not sure what supports were there from the Heart Institute, because if there is no sustainability, you are just basically saying, Okay, add this to your workload and although you mentioned great that smoking cessation is important, it is an extra item that we are expecting nurses to remember to do, one, and that they will complete, have the discussion about the IVR afterwards, and follow-up in the community.' (DM 6)

When asked if they could envision a time without support from UOHI, many respondents described the role that they felt UOHI could take in sustaining the program. '[UOHI is an] excellent link for us...gives me new research' (SCC 1). 'It is easier to keep a program going if a central institution is involved; it keeps the program on the front burner' (DM 2). Other roles a centralized institution might consider included: offering a mini-refresher course to ensure that everyone knows the newest information available; coordinating various hospital sites to ensure that information is consistent across hospitals; organizing a community of practice teleconference every two to three months between sites so that they could learn from each other; and assisting hospitals with training and resources to manage and process program statistics.

### Quality cycle - how the OMSC program demonstrates a positive impact on the health of the target population

Respondents cited the reputation and experience of the OMSC in addressing hospital smoking cessation as a major reason why they decided to implement the OMSC. The ability to demonstrate quit rates appealed to hospitals:

'It was already a success in other hospitals. They had really good evidence to support what they were doing, really good numbers [quit rates] showing how successful they had been, so in many ways it seemed like a really good model.' (SCC 2)

The best practice statement in the model was also appealing:

'It makes it easier for us to try and move the notion forward that not only were we smoke-free property-wide but that we were actually going to try and support patients while in the hospital to achieve that status of not smoking while they were a patient in the hospital.' (DM 5)

The baseline survey and other tracking measures were beneficial because they enabled hospitals to see improvement and track their progress, and increased accountability: 'People realize that the program is important because measures are reported to leadership; if they have to report it then they are held accountable' (DM 1). 'Providing feedback to staff makes them more aware of what is going on; to keep them in the loop and remind them of the processes' (SCC 1). Program results could be used to argue for funding as 'once [you] have outcomes then it becomes more sellable' (DM 2).

Hospitals used this performance feedback to make changes to their processes. When two hospitals noticed a decrease in the number of smokers being identified, one began the process to integrate a late-career nurse to provide support to the program, and the other obtained support from UOHI to develop communication tools and conduct additional training sessions. Another hospital, wanting to increase the IVR follow-up enrolment rate, now asks patients about IVR on admission and at discharge because:

'Some patients are not ready at the beginning of their stay in the hospital, but once they see how they do within the hospital then sometimes they're more open to trying to stay, to remain smoke-free. So, we would suggest the IVR again, we would ask again at the second time.' (SCC 1)

Despite the positive feedback on the measures collected by the OMSC, DMs felt that it is difficult to sustain programs that require data management without dedicated resources. One DM felt that the culture of collecting data for these types of programs has implications for their sustainability because hospitals do not have the infrastructure to collect all of this information, 'It was the reporting that was required, I'm not sure if people knew that up-front, how much reporting was expected or that they would be requested to provide' (DM 6).

### Organizational context

The OMSC program was operating within a social, political, and economic context defined by the organizational setting, community environment, and available resources. Some hospitals were challenged during implementation because collecting data and setting up the IVR component of the program involved the cooperation of different hospital departments (*e.g*., technical and privacy).

While DMs felt that the OMSC was an important initiative and had advocated for the program's implementation and continuation, they were also cognizant that smoking cessation is only one of many hospital initiatives. To avoid the program becoming forgotten amongst other new and competing initiatives, one SCC remarked that they are trying to incorporate the program into other things that the hospital is doing (*e.g*., posters for skills days), 'When you keep doing the same thing for a long time, you need to spruce it up a bit and talk about it a bit more' (SCC 1).

SCCs also noted that because nurses are busy and have competing priorities, and patients are in the hospital for shorter stays, completing a smoking assessment may not be a top priority and patients may be discharged before being offered the OMSC.

Although all DMs interviewed felt that the continuation of the OMSC depended on resources, only one hospital prepared a plan and budget for continued funding. One DM remarked that the OMSC was funded through the hospital's operating budget, but, 'It is something that I sort of have to vie for and continue to justify with my directors in terms of the hours and how that's needed' (DM 2). At another hospital, the DM reallocated funding in a specialized nursing unit which was not part of the hospital's operating budget to enable the program to continue, but only in that unit.

Study respondents identified that resources are necessary for staff education, data management, and to fund a full-time person dedicated to the OMSC. However, opinions differed as to whether assigning an overall champion or employing full-time smoking cessation counsellors would ensure that all patients receive counselling and are informed about the IVR.

Key informants remarked that the success of the program would depend on how successful hospitals are including smoking cessation as part of best practices for nurses and other health professionals. One DM suggested that for programs that aim to change behaviours, it is necessary to include these concepts in the educational curriculum of the healthcare providers to increase acceptance of the program by professionals, overcome attitudes of resistance, and to have it looked upon as an acute care health issue. Another respondent suggested that physicians become more involved in smoking cessation by talking to patients about their tobacco use prior to hospital admission.

## Discussion

This was an exploratory study to understand how hospitals using the OMSC were addressing sustainability. The OMSC was defined as sustainable if the core smoking cessation activities (identifying smokers, documenting smoking status, providing cessation advice and medication to smokers, and offering follow-up post-discharge) were performed at a higher level than when the OMSC was first implemented (baseline). Using this objective measure reduced the likelihood of misclassifying the OMSC as sustainable or not sustainable. It enabled the research team to examine similarities and differences in implementation processes, system supports and resources, and organizational culture that have been suggested to affect sustainability.

We did not find any differences in the OMSC's sustainability by hospital unit (general inpatient or special care unit), management of the IVR follow-up (hospital or UOHI), or length of time since launching the OMSC. However, we did find that hospitals with a SCC with some dedicated time (as little as 10%) to educate and train staff, promote the OMSC (either themselves or by enlisting champions), and ensure that patients are being identified, offered counselling, and follow-up had achieved OMSC activity rates that were higher than baseline. These actions may influence the sustainability of the program by enhancing the interactions between the health issue, stakeholders, and program.

Key informants identified that the UOHI training, education, and research updates should be considered a key component to the program's sustainability in a hospital setting. Both SCCs and DMs noted the need for continuous training updates given staff turnover in nursing units. Training and education provide the skills necessary to administer the program, and an opportunity to change stakeholder awareness and attitudes about hospital based cessation programs. Education about the effectiveness of patient follow-up on smoking cessation may impact the hospital's decision to continue with that component of the program.

Hospitals with a sustainable OMSC had designated SCC time for staff education, training, and support, which is consistent with Greenhalgh *et al*. [18] who found that providing staff with clear training materials and timely training opportunities enhance implementation and sustainability.

Many key informants identified the need for a passionate champion to move the issue forward and to advocate for the OMSC program. Program champions may be needed at various levels within an organization, and their message may need to be tailored to different stakeholder expectations [17,18,21]. While Scheirer [15] recommends that program developers 'identify and support' champions, Greenhalgh *et al*. [18] found few studies on this topic. Further research is needed to define the role of program champions, to understand what characteristics successful champions have, and what actions they take to enhance the sustainability processes (*e.g*., how they influence drivers).

Blasinsky *et al*. [23] examined the sustainability of a depression care management program and found at four of five sites that the ability to demonstrate positive patient outcomes was identified as the most important factor that contributed to program continuation and integration into existing systems. The OMSC uses the IVR system to track smokers after discharge and to collect, store, and report performance data. Because information on program effectiveness can enhance stakeholders' perceptions of the program's value, or prompt actions to improve performance, it is important to recognize barriers (*e.g*., staff time and actual costs) for some hospitals. Lack of performance feedback and data on cessation rates may jeopardize the sustainability of the hospital based smoking cessation interventions.

Key informants suggested that UOHI could provide support in sustaining a hospital smoking cessation program by creating communities of practice and providing up-to-date research findings and ongoing training. UOHI may also want to consider managing the IVR follow-up and performance feedback system. Future research is needed to determine the type and amount of external support that is beneficial in sustaining hospital participation yet is affordable and feasible for the supporting organization.

All key informants felt that dedicated funding was necessary for the sustainability of the OMSC; this is consistent with the review by Greenhalgh *et al*. [18], which found that programs that receive dedicated and ongoing implementation funding are more likely to be sustained. Conversely, Lapelle *et al*. [20] found community-based tobacco treatment programs that were able to find new funding, adjust staff, and create a demand for services after implementation funding was discontinued, were able to sustain services and at a higher level. Although the OMSC program components work well in the hospital setting, funding for SCC time to support the program (*e.g*., training, education, communicating results) and the IVR system represent new expenses specific to the OMSC, and hospitals were concerned with funding this supporting infrastructure. Our study was not designed to examine when new programs should become self-funding or whether some functions should be centralized and serve many hospitals. These are important research questions if effective programs like the OMSC are to become routine hospital practice.

### Limitations and strengths

#### Strengths

We distinguished between sustainable and unsustainable smoking cessation interventions by using a measurable definition of sustainability based on hospitals' performance of the OMSC intervention relative to baseline. This facilitated investigation of similarities and differences between hospitals to examine components of the OMSC and how they were being sustained. It was also possible to examine factors that have been associated with sustainability in the literature that emerged from our analysis.

Although we interviewed only two key informants per hospital, each provided similar responses to the interview questions and held similar perceptions of OMSC sustainability, organizational culture, and the value of performance feedback. Both within, and between hospitals, similar factors were identified that can affect sustainability, such as problem definition, role of program drivers, use of champions, and performance feedback. This similarity provides some validation of the study findings, and is consistent with the interactional model proposed by Gruen *et al*. [21].

#### Limitations

The exploratory nature of this study and the small number of hospitals and key informants interviewed means our findings cannot be generalized beyond those interviewed. Generalization was not the purpose of our study; rather, we sought an understanding of how hospitals were approaching sustainability. A larger study with more hospitals (especially lower OMSC activity hospitals) may lead to different conclusions. However, consistent themes emerged from this analysis that provides direction for further research.

Hospitals were selected based on UOHI records of performance, availability, and willingness to participate. We collected only limited information on the organizational characteristics of participating hospitals that could affect sustainability, particularly if competing priorities, infrastructure, or procedures directly affect sustainability. It was interesting to hear from DMs that smoking cessation is perceived to be more of a public health intervention than an acute care treatment. This needs further exploration because hospitals do implement cessation interventions when defined as a hospital problem.

As in all studies, there is the potential for a social desirability bias. Because the interviewer was not a member of the UOHI, respondents may have felt more at ease to discuss any concerns with the OMSC. The focus on understanding and open-ended nature of the interview likely reduced any concerns about an evaluative purpose of the study.

## Conclusions

The OMSC is an effective smoking cessation intervention for the hospital setting that can reduce the prevalence of smoking in the population. Success of the program is dependent upon the ability of hospitals to sustain the program in the clinical setting over time, despite competing priorities. Understanding DMs' priorities and frame of reference and showing how the intervention meets the needs of various stakeholders may impact the willingness of these drivers to prioritize the program. Using program champions, incorporating relevant performance feedback, conducting ongoing education, training, and promotion, designating a hospital based coordinator role, and demonstrating program effectiveness emerged as important factors for sustainability of the OMSC. Hospitals in this study also identified the need for centralized roles such as research updates, shared learning and potentially program monitoring and performance feedback.

In order to impact a program's sustainability it is necessary to understand the factors involved in continuing the program and to develop an approach to address any concerns [16]. This is important because 'many interventions that are found to be effective in health services research studies fail to translate into meaningful patient care outcomes across multiple contexts due to barriers at different levels within the organization' [24]. Current theories of implementation and sustainability provide a basis for further study. Gruen *et al*.'s [21] model is among the first to highlight the complex interactions between programs, health issues, and stakeholders. Hospitals that recognize and respond to these interactions may be able to sustain new programs more readily. Our findings, albeit tentative, highlight the potential importance of interactions that occur within the hospital context during program implementation. Additional model testing and clear definitions of sustainability are needed so that researchers can understand what is being measured and how different components interact with one another.

## Competing interests

RDR has received a speaker's honoraria from Pfizer, Inc. All other authors have stated no competing interests.

## Authors' contributions

SC (Lead Investigator) and KP (Lead Evaluator) were responsible for the design, conduct, analysis, and interpretation of this exploratory sustainability study of the Ottawa Model of Smoking Cessation. Both participated equally in writing this manuscript. KAM is responsible for overseeing implementation of the OMSC in Ontario hospitals. She facilitated the evaluation process, provided feedback on data interpretation and findings, and contributed to the manuscript sections on the Ottawa Model. RR participated in the design of the study and provided critical feedback to both the evaluation report and manuscript. RDR is the principal investigator of the OMSC implementation and dissemination study. He participated in the design of this study, reviewed findings and contributed to the manuscript preparation and revision. All authors have read and approved the final manuscript.

## Supplementary Material

Additional file 1 **Interview Guide**. Questions used to gain an understanding of the sustainability of the OMSC. Click here for file

Additional file 2 **Aspects of program delivery**. Overview of how different hospitals implemented the OMSC. Click here for file
