# Supplementary material for: Examining sustainability in a hospital setting: Case of smoking cessation
Source: Implement Sci. 2011 Sep 14;6:108. doi: 10.1186/1748-5908-6-108 (PMC3184081; doi:10.1186/1748-5908-6-108)
Supplement: Additional file 1 — Interview Guide. Questions used to gain an understanding of the sustainability of the OMSC. [file 1748-5908-6-108-S1.DOC]

## Interview Instrument

1. What was your role in implementing the Ottawa Model?

Probe history:

- How did the program come about? (Why did the hospital decide to implement the model?)
- Did the program start out as a pilot?
- Did you feel sufficiently informed about what the Model would do and how to implement it?
- Why did the hospital decide to implement the model on the units it did? (DM only)

Probe start up:

- Could you describe any challenges in getting started and how they were dealt with?
- How is your hospital providing support for the program (DM only)

Probe changes in operations:

- Could you describe any changes in operations?
- Why were changes made?

Factors of interest

- Organizational culture
- Decentralized or centralized decision-making (CEO mandated or let units decide)
- Fit with objectives of hospital (innovation-context fit); clear what the model would do for the hospital
- Slack resources—did invitation come at time when had extra resources (to implement?)
- Attitudes (feasible, affordable, acceptable, goals of the organization)
- Does knowing how to implement the Model make it more sustainable because it is easier to get started?

Probe role of external facilitator (SCC only)

- How important was the external facilitator in the early days? Did this change as you got more experience? Can you envision a time when the program runs with hospital staff providing feedback etc. (i.e. no external facilitators)?
- Did commitment to program diminish when external facilitator time was less?

Factors of Interest (SCC only)

- Early use—continuing access to information, training, support
- How critical is the external facilitator to sustainability?

Probe changes/adaptations made:

- How has your role changed? What was your role and how did it change?
- Describe any changes /adaptations to the Model? (Let respondent identify changes made)
- If no changes, why?

Probe Implementation Expectations

- What were expectations about the program when it started?
- Always have a cessation coordinator or that all nursing staff would do counselling as part of routine care?
- How has it gone (based on expectations)?

Probe reasons adaptation made:

- Why were these changes made?
- What other options were considered?
- Why was that option chosen? How did it work? What worked and what didn’t?

Probe who made decisions

- Who made the decision to make the change?

1. What are the costs the hospital is expending to implement the model?

Probes

- Is the hospital covering IVR costs?
- How were costs approached?
- Were costs within the planned estimates?

**Summative**

Is there anything else that you would like to elaborate on or share regarding the program’s history and its implementation?

1. Do you feel that the Ottawa Model is part of hospital routine now? Why or why not?
2. What do you see as the future of the Ottawa Model for your hospital? (What will the program look like in 2 years, in 5 years?)

Prompts:

- How confident are you that the program will be continued sustained in your hospital a year or 5 years from now? Why are you confident or not confident? (i.e., What factors helped form this opinion?)
- Which parts of the program would be continued, why and why not?
- Describe any changes you foresee in the program’s future.
- What do you consider as realistic or acceptable in terms of identifying smokers, counselling, and IVR? Are you achieving this rate? Why or why not? (SCC only)

Probe challenges/barriers and positives/helpers to sustaining OMSC:

- What makes the OMSC easy/difficult to sustain? Challenges?
- What do you think will be the greatest barriers (threats) to other sites or organizations adopting the program?
- Describe any systems your organization have a system in place for overcoming these barriers?
- Will additional funding be needed?
- Would you choose to implement the Ottawa Model again? Why, why not? What would you do differently?
- Does having the support from a central institution make it easier to sustain a program? (DM only)

1. Are there any documents that would provide us with any more additional information about the implementation of the OMSC or the decision to continue with the OMSC? (i.e. policies, discussion documents about hospital role in smoking cessation, or to continue with the OMSC)
2. What is the lifespan of new programs like this one? (DM only) (We will try to place the responses into the context of comparing to other new programs)
3. What is your current role? How long have you been in your current role?

**Summative**

Is there anything else that you would like to elaborate on or share regarding the program and its sustainability in your hospital?

Is there anything else you would like to discuss about the Ottawa Model?
