# Supplementary material for: Examining sustainability in a hospital setting: Case of smoking cessation
Source: Implement Sci. 2011 Sep 14;6:108. doi: 10.1186/1748-5908-6-108 (PMC3184081; doi:10.1186/1748-5908-6-108)
Supplement: Additional file 2 — Aspects of program delivery. Overview of how different hospitals implemented the OMSC. [file 1748-5908-6-108-S2.DOC]

**Additional file 2: Table S1. Aspects of program delivery**

|  | **Level of OMSC Activity** | **Date Launched** | **Program Location** | **Smoking Cessation Coordinator Responsibilities** | **Who Delivers Program** | **Who Monitors IVR** |
| --- | --- | --- | --- | --- | --- | --- |
| Hospital A | higher | 2006 | all inpatient units | -nurse spends 10% of her time on smoking cessation  -OMSC specific: train new staff, present at staff meetings on ad hoc basis | -nurses as part of patient care | UOHI |
| Hospital B | higher | 2007 | all inpatient units | -nurse has four hours per week to counsel patients, liaise with doctors and enter patients into IVR | -all nurses identify patients  -five nurses trained to counsel patients | UOHI |
| Hospital C | higher | 2007 | all (*i.e.,* four) units within a particular program | -nurse is given 50% of her time for nursing and administrative support (staff training, liaise with smoking champions in other units, enter patients into IVR system, follow-up with IVR patients, provide feedback to nurses, provide information to administration) | -nurses as part of patient care | hospital |
| Hospital D | higher | 2008 | select units; in the process of expanding to other units | - full time role to supervise nurse counselor, develop tools for staff education, public relations (give OMSC a profile in hospital) | -nurses identify smokers  -dedicated counselors deliver the program | hospital |
| Hospital E | lower | 2008 | in a specialized program | -IVR  -integrated with other program duties | -dedicated counselors  -in other units smokers may be identified by nurses and NRT may be prescribed | hospital |
| Hospital F | lower | 2007 | all inpatient units | -no smoking cessation coordinator  -exploring the possibility of a late-career nurse to assist with this program (details of position not determined) | -all nurses identify smokers and are to deliver the program  -in practice, four nurses have received training from UOHI  -team leader may do some monitoring if time permits | UOHI |
